# Artificial Intelligence to Determine Correct Midsagittal Plane in Dynamic Transperineal Ultrasound

**DOI:** 10.1002/jcu.24050

**Published:** 2025-04-25

**Authors:** José Antonio García‐Mejido, Juan Galán‐Paez, David Solis‐Martín, Marina Martín‐Morán, Carlota Borrero‐Gonzalez, Alfonso Fernández‐Gomez, Fernando Fernández‐Palacín, José Antonio Sainz‐Bueno

**Affiliations:** ^1^ Department of Surgery, Faculty of Medicine University of Seville Seville Spain; ^2^ Department of Computer Science and Artificial Intelligence, Faculty of Mathematics University of Seville Seville Spain; ^3^ Department of Statistics and Operational Research University of Cadiz Cadiz Spain

**Keywords:** artificial intelligence, gradient boosting, levator ani muscle, machine learning, pelvic floor, ultrasonography, XGBoost

## Abstract

**Purpose:**

To create and validate a machine learning(ML) model that allows for identifying the correct capture of the midsagittal plane in a dynamic ultrasound study, as well as establishing its concordance with a senior explorer and a junior explorer.

**Methods:**

Observational and prospective study with 90 patients without pelvic floor pathology. Each patient was given an ultrasound video where the midsagittal plane of the pelvic floor was recorded at rest and during the Valsalva maneuver. A segmentation model was used that was trained on a previously published article, generating the segmentations of the 90 new videos to create the model. The algorithm selected to build the model in this project was XGBoost(Gradient Boosting). To obtain a tabular dataset on which to train the model, feature engineering was carried out on the raw segmentation data. The concordance of the model, of a junior examiner and a senior examiner, with the expert examiner was studied using the kappa index.

**Results:**

The first 60 videos were used to train the model and the last 30 videos were reserved for the test set. The model presented a kappa index 0.930(*p* < 0.001) with very good agreement for detection of the correct midsagittal plane. The junior explorer presented a very good agreement (kappa index = 0.930(*p* < 0.001)). The senior explorer presented a kappa index 0.789(*p* < 0.001) (good agreement) for detection of the correct midsagittal plane.

**Conclusion:**

We have developed a model that allows determining the correct midsagittal plane captured through dynamic transperineal ultrasound with a level of agreement comparable to or greater than that of a junior or senior examiner, using expert examiner assessment as the gold standard.

## Graphical abstract

We have created a model that allows determining the correct midsagittal plane captured by transperineal dynamic ultrasound, with a level of agreement with the expert examiner similar to or higher than that of a junior or senior examiner.

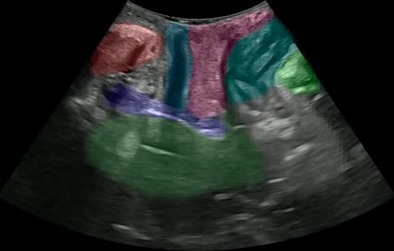

## 1 Introduction

Pelvic floor ultrasound has represented a breakthrough in the study and diagnosis of pelvic floor dysfunctions. One of the characteristics of transperineal pelvic floor ultrasound is that it is standardized from the midsagittal plane (García‐Mejido, Bonomi‐Barby, et al. 2020) to two‐dimensional ultrasound. From the midsagittal plane, we can simultaneously study the pubic symphysis, the urethra, the urinary bladder, the vagina, the uterus, the anal canal, the rectum, and the levator ani muscle (García‐Mejido, Bonomi‐Barby, et al. 2020) and therefore most of the pathology that covers each compartment. Correct capture of the midsagittal plane requires learning that sometimes depends on the skills of the examiner. In addition, transperineal ultrasound is associated with manual measurements, which involve a time consumption in the consultation and depend on the experience of the examiner, which will directly influence the variations in the score (Thyer et al. 2008). To these aspects, we must add that pelvic floor ultrasound allows a dynamic study of the different structures, making its study more complicated, especially in the case of pelvic organ prolapse. In fact, it has been described that in order not to lose the midsagittal plane during the Valsalva maneuver, the rotational movement of the transducer must be avoided, preserving the original alignment of the pelvic floor in the ultrasound image (Shek and Dietz 2015).

On the other hand, the development of artificial intelligence (AI) in the field of urogynecology is progressively expanding, showing its usefulness in identifying different structures of the pelvic floor (Huang and Chen 2007; van den Noort et al. 2019, 2018; Bonmati et al. 2018). AI allows a computer program to perform reasoning processes similar to the human brain, with deep learning (DL) being a subcategory that can recognize medical images, classify them, and detect objects (Vianna et al. 2021). Currently, CNN (convolutional neural network) has been used in pelvic floor ultrasound for the analysis of the levator ani muscle (van den Noort et al. 2019; Vianna et al. 2021; Feng et al. 2020; Rabbat et al. 2023), for measurements of the levator hiatus (van den Noort et al. 2019; Bonmati et al. 2018; Huang et al. 2022; Li et al. 2019), measurement of the urogenital hiatus (FvD et al. 2022), the assessment of urodynamic stress incontinence with ultrasound (Huang and Chen 2007) and for the study of pelvic organ prolapse (Duan et al. 2021). However, all these studies are based on static images, and to obtain the ultrasound diagnosis of the different pelvic floor dysfunctions, we need a dynamic ultrasound study (Shek and Dietz 2015). Recently, it has been described that it is possible to apply deep learning to identify the different pelvic floor organs in a dynamic ultrasound study in the correctly captured midsagittal plane (García‐Mejido et al. 2024). However, sometimes it is possible that the midsagittal plane is not well defined, either due to the condition of the patient's tissues or due to the lack of training of the examiner who performs the technique. These defects in the capture of the image can lead to diagnostic errors and interpretation of the ultrasound. Based on these aspects, we consider that AI can be of great help if it can help us define the correct midsagittal plane for the ultrasound study of the pelvic floor. Therefore, our objective is to create and validate a predictive model that allows us to identify the correct midsagittal plane in a dynamic ultrasound study, as well as establish its concordance with a senior examiner and a junior examiner.

## 2 Materials

An observational and prospective study was conducted with 90 patients. The included patients had no pelvic floor pathology and were recruited consecutively in the general gynecology clinic from May 1, 2024 to June 31, 2024. Patients with difficulty performing the Valsalva maneuver or a history of pelvic floor dysfunction were excluded. The following clinical parameters were collected for each patient: age, weight, height, body mass index (BMI), parity, menopausal status, age at menopause.

### 2.1 Ultrasound Examination

**FIGURE 1:**
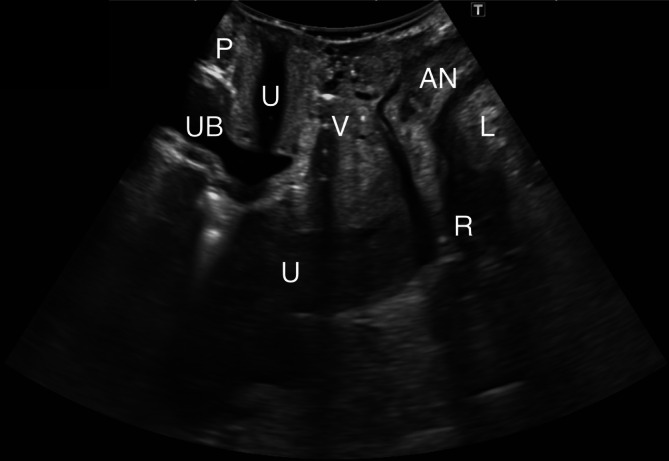
Shows the correct midsagittal plane. Pubis (P), urethra (U), urinary bladder (UB), vagina (V), uterus (U), anus (AN), rectum (R), levator ani muscle (L).

**FIGURE 2:**
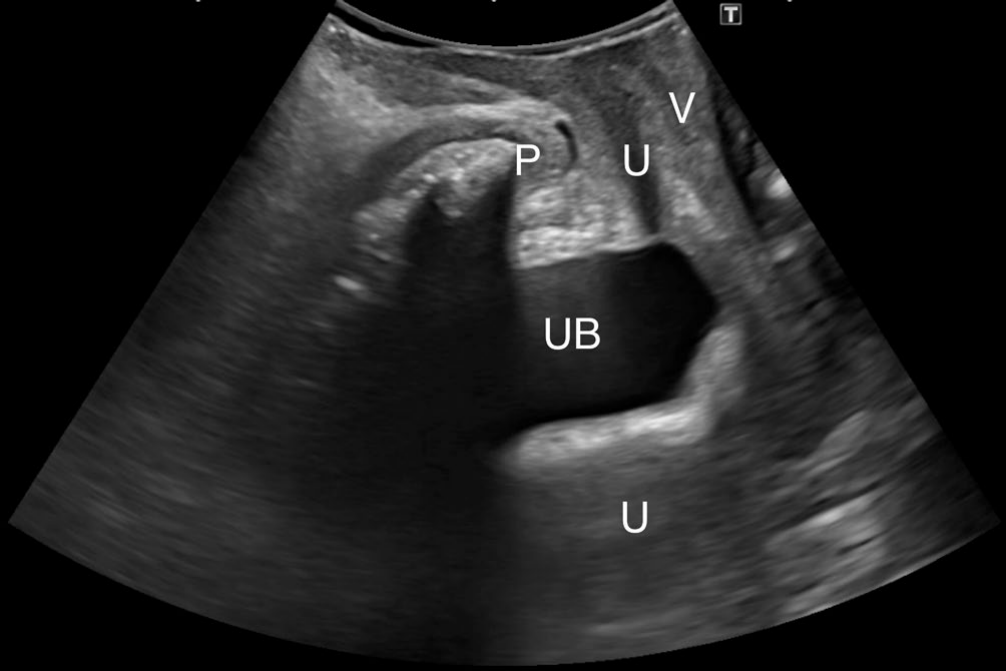
Incorrect midsagittal plane where the anus, rectum, and levator ani muscle are not visualized due to displacement in the sagittal axis. Pubis (P), urethra (U), urinary bladder (UB), vagina (V), uterus (U).

**FIGURE 3:**
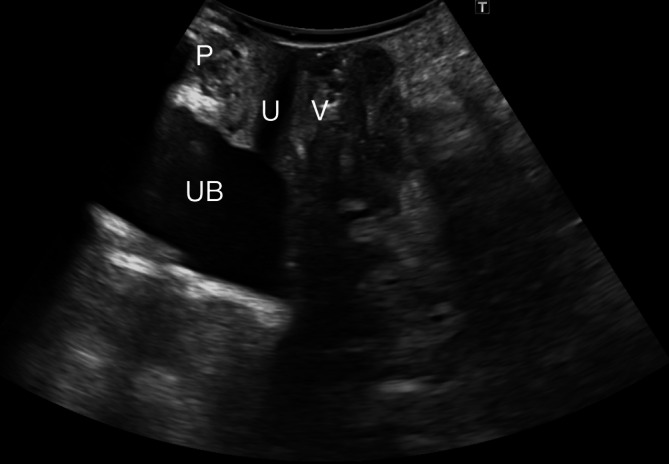
Incorrect midsagittal plane where the uterus, anus, rectum, and levator ani muscle are not visualized due to a rotation of the image (C). Pubis (P), urethra (U), urinary bladder (UB), vagina (V).

All transperineal ultrasounds were performed by the same expert pelvic floor ultrasound examiner using a Canon i700 Aplio (Canon Medical Systems Corp., Tokyo, Japan) ultrasound with a PVT‐675MV 3D abdominal probe. Images were acquired following guidelines previously established in the literature, with patients in dorsal lithotomy position with hips flexed (García‐Mejido, Bonomi‐Barby, et al. 2020). Prior to capturing and storing the video, the patient was trained to correctly perform the Valsalva maneuver. Each patient was given an ultrasound video capture showing the midsagittal plane of the pelvic floor at rest and the Valsalva maneuver. The ultrasound videos were oriented by placing the cranioventral region on the left and the dorsocaudal region on the right. The expert examiner captured 45 videos of the correct midsagittal plane and 45 videos of the incorrect midsagittal plane. A correct midsagittal plane was defined as one that included the view of the pubic symphysis, urethra, bladder, vagina, uterus, anus, rectum, and levator ani muscle (Figure 1). An incorrect midsagittal plane was defined when any of the previously described anatomical structures was missing, either due to a displacement of the probe in the anteroposterior axis (Figure 2) or a rotation of the image (Figure 3).

### 2.2 Algorithm

The aim of this work is to build a model that determines whether the ultrasound was performed correctly for each specific organ. In order to create such a model, the first 60 videos were used as the training set, whereas the last 30 cases were reserved as the test set.

**FIGURE 4:**
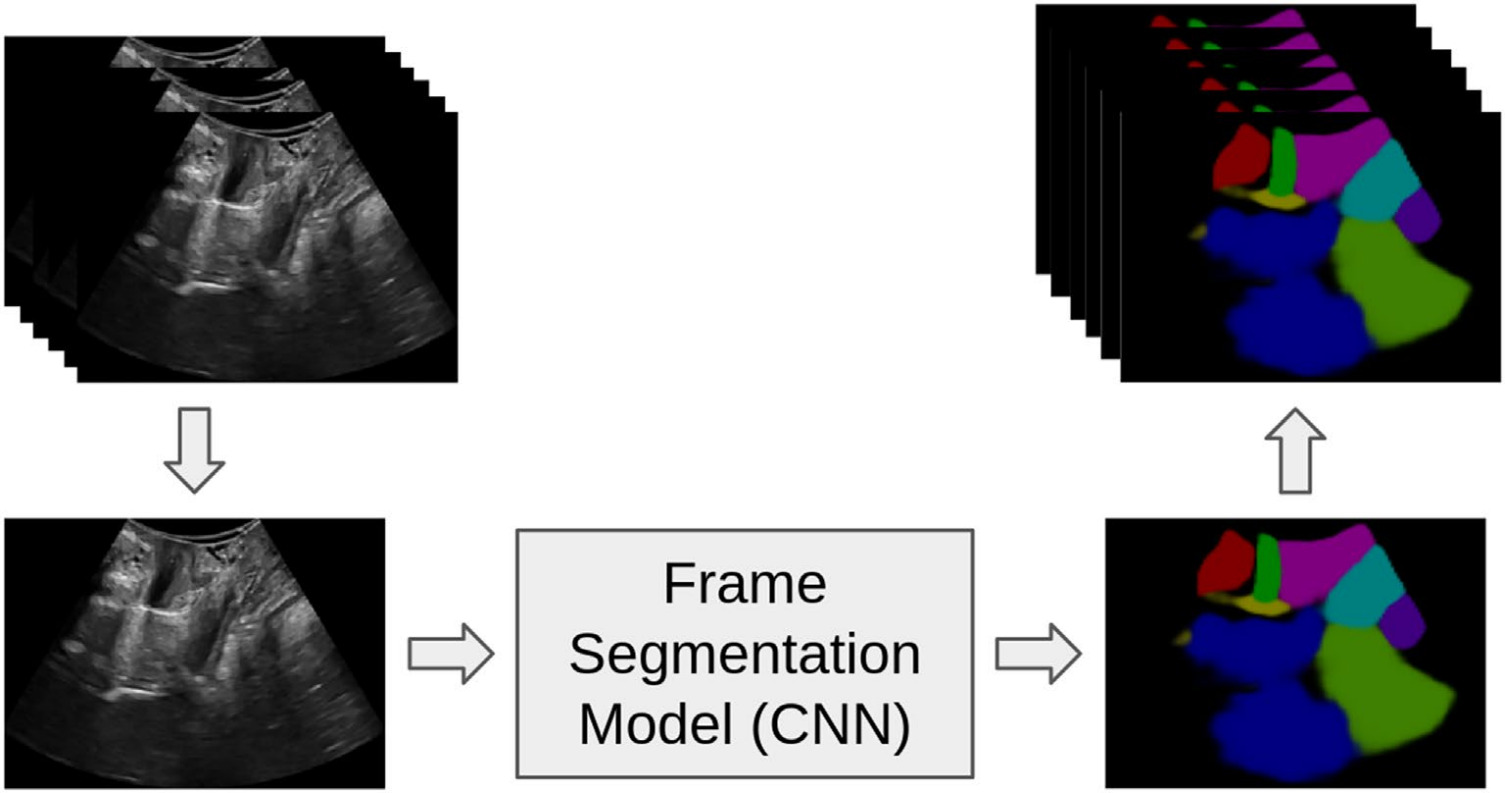
Extraction of frames from the video and sending to the segmentation model that will generate segmentation by image.

For this project, a segmentation model trained on a previously published paper (García‐Mejido et al. 2024), that aims to identify organ positions on images (frames) extracted from ultrasound videos, was used to generate segmentations of the 90 new videos. In this stage, the video frames are extracted and sent one by one to the mentioned segmentation model, which will generate a segmentation per image (Figure 4).

From the segmentations, a set of features or statistics was extracted for each frame and each segmented organ to capture the relative confidence in the identification of each organ. The extracted features include the average confidence level of the full segmentation (prediction mean), confidence variation of the full segmentation (prediction deviation), maximum confidence level of the full segmentation (maximum prediction value), and minimum confidence level of the full segmentation (minimum prediction value).

Additionally, for each organ in each frame, these same features were calculated, but only considering prediction values greater than 0.5. This approach allows capturing the absolute confidence of the model in the identification of the organs, compared to the general confidence in each frame.

If the values obtained for the total predictions are significantly lower than those calculated for predictions greater than 0.5, it indicates that the confidence assigned to the pixels that do not belong to the organ is very low, reflecting a high confidence of the model in the identification of the organ. Conversely, if the values between both groups are similar, it means that high levels of confidence have been assigned to pixels outside the organ, indicating lower certainty of the model in the segmentation.

For each segmentation of each organ, its bounding box was calculated, i.e., the box that contains the predicted segmentation. For each bounding box, the centroid, the maximum and minimum coordinates on each axis, the width, height, and area were calculated. The rationale for these features was that the model could determine whether the location, size, and aspect ratio were consistent for that organ.

**FIGURE 5:**
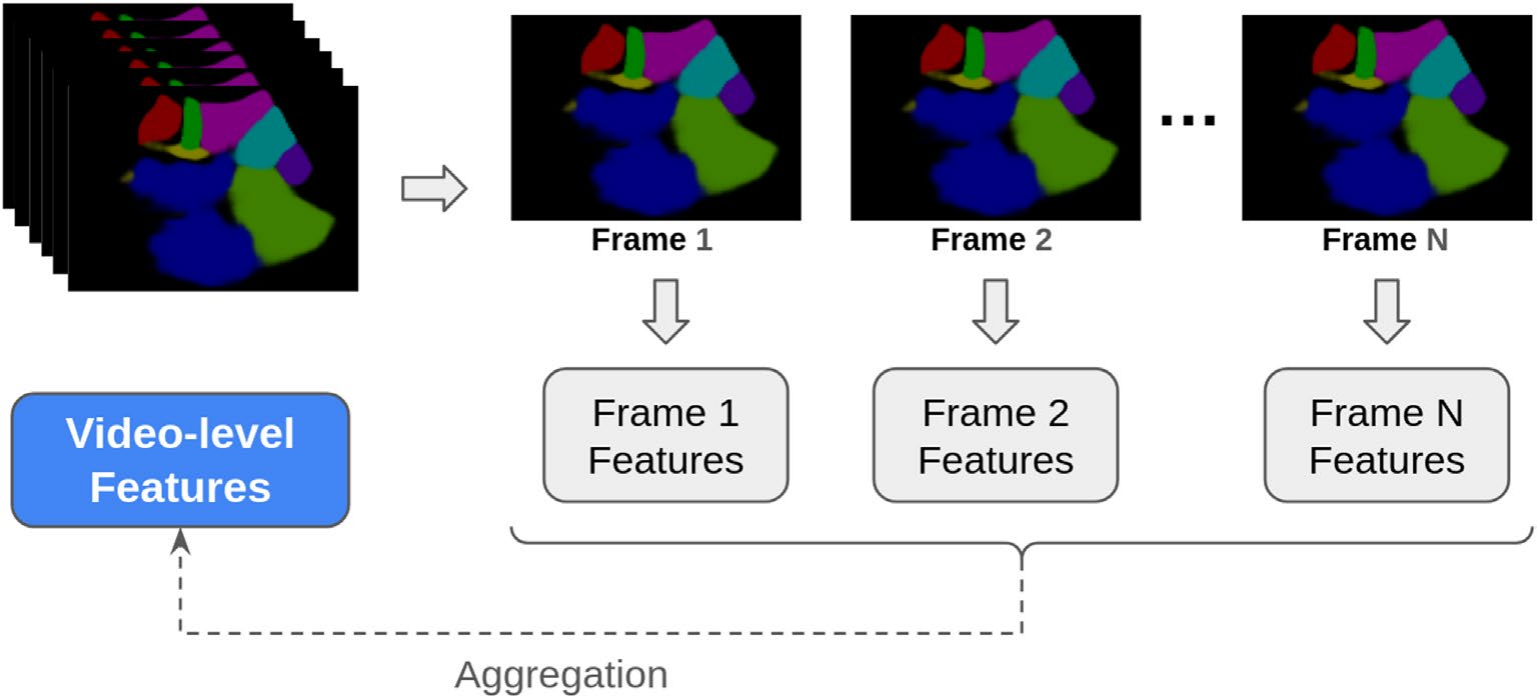
Aggregation of computed features at the frame sequence level.

To build a tabular dataset in which each row represents a patient and an organ, labeled as “correct” or “incorrect,” it was necessary to aggregate the features calculated at the frame level to the patient or ultrasound level, i.e., at the frame sequence level (Figure 5). This aggregation was performed by calculating the mean, standard deviation, maximum value, and minimum value of the features obtained over a sequence of N frames.

**FIGURE 6:**
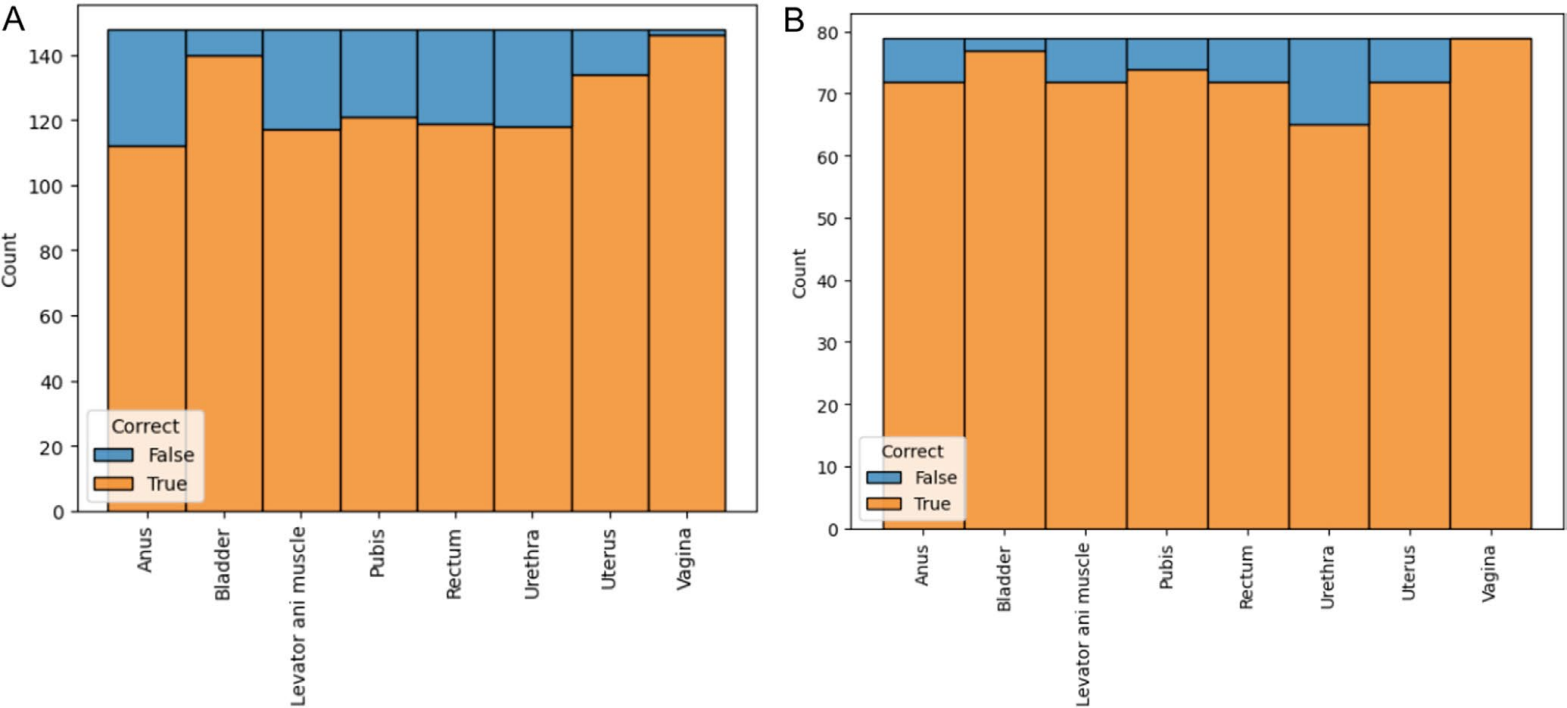
Train set distribution (A) and test set distribution (B).

As a result, a dataset was generated in which each row represents an organ and a sequence of N frames, with a total of 68 features per row. This dataset was used to train a model that determines whether the ultrasound was performed correctly for each specific organ. *N* = 60 was set, resulting in a total of 1816 rows for the 90 cases analyzed. After dividing the set into training and test subsets, 1184 rows were obtained for the training set and 632 for the test set. Figure 6 shows the distribution of the samples in both subsets.

The value of *N* = 60 was chosen instead of using the complete data sequence of each ultrasound as a “data augmentation” technique, useful when there is little data available. If only one row per ultrasound had been generated, the dataset would have 720 rows, given that there are 90 patients and 8 organs per patient. However, by using windows of N frames, multiple rows were generated per patient and organ, which increased the dataset's size to 1816 rows.

**FIGURE 7:**
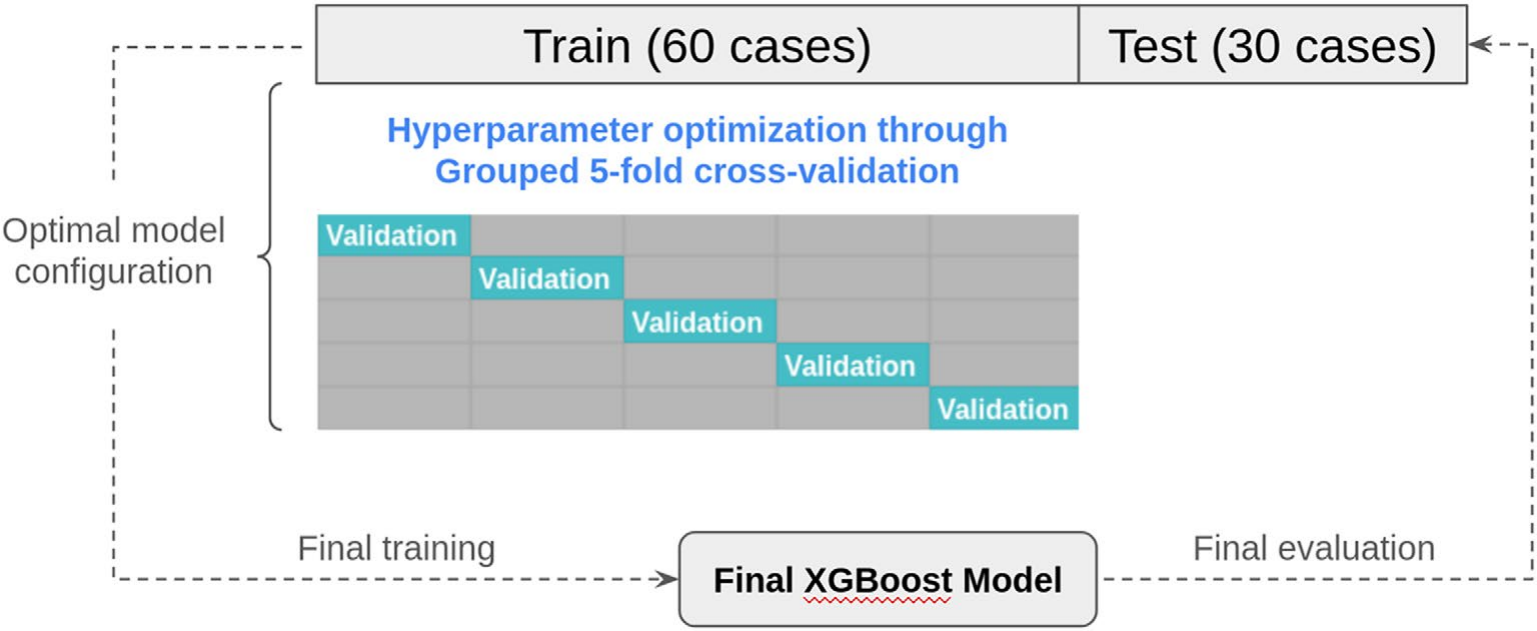
Hyperparameter optimization through grouped 5‐fold cross‐validation.

At the end of this stage, the final dataset had been built and split into training and test datasets. In order to obtain the best model, a hyperparameter optimization process (random search) was executed on the training set. Since there are multiple rows that belong to the same patient, model evaluation during the optimization process was carried out using grouped k‐fold cross‐validation to avoid data leakage. Once an optimal model configuration has been obtained, the final model is trained using the whole training set and evaluated on the reserved test set, that is, the last 30 cases (Figure 7).

The ML algorithms considered in this work fall within decision tree‐based methods. Specifically, the algorithm used to obtain the final model was XGBoost (Chen and Guestrin 2016) (Gradient Boosting (Gradient Boosting and Friedman 2001)), as it has shown to obtain the best results so far. Although the performance of Random Forest was also evaluated, XGBoost outperformed it in all tests performed.

### 2.3 Concordance With the Expert Examiner

The assessment of the expert examiner in pelvic floor ultrasound (JAGM, a reference ultrasonographer at their hospital with over 10 years of experience in pelvic floor ultrasound) was used as the gold standard. The expert examiner determined whether the video captured the correct or incorrect midsagittal plane and also identified which organs were properly visualized for interpretation. Based on these determinations made by the expert examiner and using them as the gold standard, the concordance of the model was analyzed alongside that of a junior examiner (a final‐year resident with knowledge of pelvic floor ultrasound anatomy but no practical experience) and a senior examiner (a gynecologist with over 15 years of experience in gynecological ultrasound). The concordance was studied in 30 recent videos captured by the expert examiner (19 correct midsagittal planes and 11 incorrect midsagittal planes). Both the model, the junior examiner, and the senior examiner had to determine whether the image captured by the expert examiner had been captured correctly or not, and to establish which types of organs were well captured in the provided video.

## 3 Statistical Analysis

The statistical analysis was performed using IBM SPSS Statistics version 26 (IBM, Armonk, NY). Data were reviewed prior to the statistical analysis. Means and standard deviations (SD) were used to describe numerical variables, while medians and percentiles (p25 and p75) were used in cases of asymmetric distribution. Qualitative variables were expressed as percentages.

The agreement between the expert examiner with the model, the junior examiner, and the senior examiner was evaluated using Cohen's kappa coefficient and its 95% CI, determined as follows: poor agreement < 0.20, weak agreement between 0.21 and 0.40, moderate agreement between 0.41 and 0.60, good agreement between 0.61 and 0.80, and very good agreement > 0.81.

## 4 Results

90 patients without pelvic floor pathology were included. The first 60 videos were used to train the predictive model, and the last 30 videos were reserved for the test set. The clinical characteristics of the 30 patients included in the test set are shown in Table 1.

**TABLE 1:** Characteristics of the patients included in the test set.

	*n* = 30	95% CI
Age	46.1 ± 13.9	40.9; 51.3
Weight	68.9 ± 10.1	65.1; 72.6
Height	161.5 ± 5.9	159.3; 163.7
BMI	26.3 ± 4.3	24.6; 27.9
Parity	1.5 ± 1.1	1.1; 1.9
Menopause	9 (31.0%)	17.1%; 49.4%
Menopause age	53.7 ± 1.5	52.5: 54.8

Table 2 shows the agreement between the model and the expert examiner, with the kappa index ranging from 0.516 (*p* < 0.001) to 1.0 (p < 0.001). The model presented a kappa index of 0.930 (*p* < 0.001), indicating very good agreement for detecting the correct midsagittal plane. Regarding the assessment of different pelvic organs, we observed that the CNN showed very good agreement for the correct visualization of the pubis (kappa index 1.0, *p* < 0.001), urethra (kappa index 1.0, *p* < 0.001), bladder (kappa index 1.0, *p* < 0.001), and levator ani muscle (kappa index 0.870 (*p* < 0.001)). There was good agreement for the correct visualization of the uterus (kappa index 0.783, *p* < 0.001) and anus (kappa index 0.760 (*p* < 0.001)). There was moderate agreement for the correct visualization of the rectum (kappa index 0.516, *p* < 0.001).

**TABLE 2:** Kappa index assessment of the predictive model for the correct identification of the different pelvic floor organs using the expert examiner's examination as the gold standard.

	Predictive model (*n* = 30)	*p* (McNemar)	Kappa (*p*)
Correct midsagittal plane	19 (63.3%)	1.0	0.930 (< 0.001)
Correct visualization of the pubis	27 (90.0%)	1.0	1.0 (< 0.001)
Correct visualization of the urethra	23 (76.7%)	1.0	1.0 (< 0.001)
Correct visualization of the urinary bladder	29 (96.7%)	1.0	1.0 (< 0.001)
Correct visualization of the vagina	30 (100%)	—	— (−)
Correct visualization of the uterus	27 (90.0%)	1.0	0.783 (< 0.001)
Correct visualization of the anus	25 (83.3%)	1.0	0.760 (< 0.001)
Correct visualization of the rectum	26 (86.7%)	1.0	0.516 (< 0.001)
Correct visualization of the levator ani muscle	26 (86.7%)	1.0	0.870 (< 0.001)

Table 3 shows the agreement between the junior examiner and the expert examiner. The junior examiner presented very good agreement (kappa index 0.930, *p* < 0.001) for detecting the correct midsagittal plane. Regarding the assessment of different pelvic organs, we observed that the junior examiner showed very good agreement for the correct visualization of the urethra (kappa index 0.902, *p* < 0.001). There was good agreement for the correct visualization of the uterus (kappa index 0.651, *p* < 0.001), anus (kappa index 0.609, *p* < 0.001), rectum (kappa index 0.615, *p* < 0.001), and levator ani muscle (kappa index 0.667, *p* < 0.001). The junior examiner was unable to correctly identify the three cases where the pubis was not correctly visualized. The same issue occurred with the identification of the correct visualization of the bladder, where the agreement was worse.

**TABLE 3:** Kappa index assessment of the junior examiner for the correct identification of the different pelvic floor organs using the expert examiner's examination as the gold standard.

	Junior examiner (*n* = 30)	*p* (McNemar)	Kappa (*p*)
Correct midsagittal plane	19 (63.3%)	1.0	0.930 (< 0.001)
Correct visualization of the pubis	30 (100%)	—	— (—)
Correct visualization of the urethra	24 (80.0%)	1.0	0.902 (< 0.001)
Correct visualization of the urinary bladder	28 (93.3%)	1.0	−0.047 (0.786)
Correct visualization of the vagina	30 (100%)	—	— (—)
Correct visualization of the uterus	29 (96.7%)	1.0	0.651 (< 0.001)
Correct visualization of the anus	26 (86.7%)	1.0	0.609 (< 0.001)
Correct visualization of the rectum	24 (80.0%)	0.250	0.615 (< 0.001)
Correct visualization of the levator ani muscle	24 (80.0%)	1.0	0.667 (< 0.001)

Table 4 shows the agreement between the senior examiner and the expert examiner. The senior examiner presented a kappa index of 0.789 (*p* < 0.001) (good agreement) for detecting the correct midsagittal plane. Regarding the assessment of different pelvic organs, we observed that the senior examiner showed very good agreement for the correct visualization of the urethra (kappa index 0.902, *p* < 0.001). There was good agreement for the correct visualization of the anus (kappa index 0.609, *p* < 0.001) and the levator ani muscle (kappa index 0.667, *p* < 0.001). There was moderate agreement for the correct visualization of the uterus (kappa index 0.444, *p* < 0.001) and rectum (kappa index 0.429, *p* < 0.001). The senior examiner, like the junior examiner, was unable to correctly identify when the pubis was not correctly visualized, presenting weak agreement with the expert examiner (kappa index 0.348, *p* = 0.051). The senior examiner also failed to detect the case with incorrect bladder visualization.

**TABLE 4:** Kappa index assessment of the senior examiner for the correct identification of the different pelvic floor organs using the expert examiner's examination as the gold standard.

	Senior examiner (*n* = 30)	*p* (McNemar)	Kappa (*p*)
Correct midsagittal plane	19 (63.3%)	1.0	0.789 (< 0.001)
Correct visualization of the pubis	28 (93.3%)	1.0	0.348 (0.051)
Correct visualization of the urethra	24 (80.0%)	1.0	0.902 (< 0.001)
Correct visualization of the urinary bladder	30 (100%)	—	— (—)
Correct visualization of the vagina	30 (100%)	—	— (—)
Correct visualization of the uterus	24 (80.0%)	0.125	0.444 (0.003)
Correct visualization of the anus	26 (86.7%)	1.0	0.609 (< 0.001)
Correct visualization of the rectum	25 (83.3%)	0.625	0.429 (0.014)
Correct visualization of the levator ani muscle	24 (80.0%)	1.0	0.667 (< 0.001)

## 5 Discussion

We have designed a model that allows identifying a correct midsagittal plane for the dynamic ultrasound study of the pelvic floor, offering a very good level of agreement with the image captured by the expert examiner (kappa index = 0.930, *p* < 0.001). This agreement coincides with that presented by the junior examiner. However, when evaluating the kappa index of the senior examiner for identifying the correct midsagittal plane, we observed that the kappa index is lower (kappa index = 0.789, *p* < 0.001). This aspect may be due to the fact that both the training of the junior examiner and the learning of the model are based on previously captured images, limited to identifying structures that are considered well captured. However, the senior examiner, unlike the model or the junior examiner, is accustomed to capturing the images they are going to interpret, and this aspect influences their ability to identify ultrasound structures in videos captured by another examiner. Similarly, we believe this aspect may also influence the identification of the correct visualization of different organs, as the model is limited to identifying different structures without being influenced by knowledge of capturing the midsagittal plane, which the junior and senior examiners possess. We consider this to be a possible explanation for why the model presents better kappa indices for the correct visualization of different organs compared to the junior and senior examiners.

Currently, a model has been described that allows dynamically identifying all the organs of the pelvic floor in the midsagittal plane (García‐Mejido et al. 2024). Additionally, in static images of the midsagittal plane, it has been possible to obtain biometric measurements with high reproducibility compared to manual analysis (Szentimrey et al. 2023). However, these works start from a correct midsagittal plane.

At the moment, the learning process for capturing the correct midsagittal plane via transperineal ultrasound is not complex (García‐Mejido, Fernández‐Palacín, et al. 2020). However, the literature does not describe an individual's ability to identify the correct midsagittal plane in a dynamic video captured by an expert examiner. This project addresses this aspect, demonstrating that a model can have an identification capability for a correct midsagittal plane similar to or superior to that of a human examiner. Incorporating this aspect into our ultrasound equipment could provide a guarantee point for diagnosing different pelvic floor dysfunctions, as it would identify the correct plane upon which we can establish different dysfunctions in the dynamic ultrasound study.

Reviewing the current literature, we observe that there are few studies on pelvic floor ultrasound that first process the ultrasound using a CNN and then apply an ML model like Gradient Boosting or Random Forests to determine a specific objective. Muta et al. (Muta et al. 2024) used Random Forests and Gradient Boosting (lightGBM) to develop a high‐precision algorithm for the automatic evaluation of pelvic floor muscle contraction from ultrasound videos. Previously, Ni et al. (Ni et al. 2016) used Random Forests to develop the first automatic solution for classifying cystocele severity in transperineal ultrasound videos.

The majority of studies on AI in pelvic floor ultrasound are based on CNNs, used to determine the area of the levator hiatus via three‐dimensional ultrasound with a dice similarity index (DSI) greater than 0.9 (van den Noort et al. 2019; Bonmati et al. 2018; Huang et al. 2022; Li et al. 2019). CNNs have also been applied for measuring the levator ani muscle, presenting DSIs ranging between 0.6 and 0.77 (van den Noort et al. 2019; Vianna et al. 2021; Feng et al. 2020; Rabbat et al. 2023). A CNN has also been described that dynamically identifies the different organs of the pelvic floor with an overall DSI of 0.79 (0.73;0.82), obtaining better results for those organs closer to the transducer (pubis, vagina, anus, and levator ani muscle) and worse results for those further away, such as the urinary bladder and uterus (García‐Mejido et al. 2024). The application of AI in pelvic floor pathology has also been defined in the literature, establishing a CNN that allows the diagnosis of pelvic organ prolapse in static ultrasound images (Duan et al. 2021). However, this technology is currently not available in routine clinical practice. Therefore, the study of pelvic organ prolapse via ultrasound is manually performed by assessing the descent of the organ in relation to the posteroinferior margin of the pubic symphysis during Valsalva (Shek and Dietz 2015), conducting differential diagnosis (Eisenberg et al. 2010; Green Jr. 1975; Chantarasorn and Dietz 2012; García‐Mejido et al. 2021, 2022a; Dietz and Steensma 2005) through measurements (Shek and Dietz 2015; Chantarasorn and Dietz 2012; García‐Mejido et al. 2021), diagnostic software (García‐Mejido et al. 2022b, 2023), or morphological differences (García Mejido et al. 2022). All this methodology has in common the need to maintain a stable midsagittal plane, using criteria to ensure a stable reference line [3], which is operator‐dependent. We present a CNN that determines the correct midsagittal plane in ultrasound, offering future studies the possibility of applying this technology to maintain the reference plane, especially in pelvic organ prolapse.

The main highlight of our study is that we establish the basis for an AI to dynamically identify the correct midsagittal plane via ultrasound, thus avoiding capture errors by the examiner. The main weakness lies in the fact that the sample size is limited and also there was only one junior examiner and one senior examiner. In addition, the test set images have few cases of incorrect visualization of certain organs, such as the urinary bladder, with only one case considered. For the model, this aspect was not an issue as it correctly identified the remaining 29 cases. However, for both the junior and senior examiners, the fact that there was only one case of incorrect bladder visualization influenced their kappa indices, as they were unable to identify this single case.

In conclusion, we have developed a model that allows determining the correct midsagittal plane captured through dynamic transperineal ultrasound with a level of agreement comparable to or greater than that of a junior or senior examiner, using expert examiner assessment as the gold standard.

## Ethics Statement

The study was approved by Andalucia's Board of Biomedicine Ethics Committee, with code SICEIA‐2024‐001928. The study was conducted in accordance with the Declaration of Helsinki.

## Consent

All patients gave their written informed consent before starting the study.

## Conflicts of Interest

The authors declare no conflicts of interest.

## Data Availability

The data that support the findings of this study are available from the corresponding author upon reasonable request.
